# Prognostic Parameters for the Primary Care of Melanoma Patients: What Is Really Risky in Melanoma?

**DOI:** 10.1155/2011/521947

**Published:** 2011-10-11

**Authors:** Daniela Göppner, Martin Leverkus

**Affiliations:** ^1^Department of Dermatology and Venereology, Otto-von-Guericke-University Hospital, Leipziger Str. 44, 39120 Magdeburg, Germany; ^2^Department of Dermatology, Venereology and Allergology, Medical Faculty Mannheim, Ruprechts Karls-University of Heidelberg, Theodor-Kutzer-Ufer 1-3, 68167 Mannheim, Germany

## Abstract

Due to intensified research in recent years, the understanding of the molecular mechanisms involved in the development of melanoma has dramatically improved. The discovery of specific, causal mutations such as BRAF or KIT oncogenes not only renders a targeted and thus more effective therapeutic approach possible, but also gives rise to a new genetic-based classification. Targeting just a few out of several potential mutations, BRAF-Inhibitors such as PLX 4032 achieved already tremendous results in the therapy of metastatic melanoma. Up to now, the correlation of clinical, histomorphologic, and genetic features is, however, not understood. Even more, is it not well known precisely what kind of molecular changes predispose the primary melanoma for metastasis. The identification of morphological surrogates and prognostic parameters in tumors with such genetic alteration seems therefore crucial when differentiating and classifying this heterogeneous tumor entity in more detail and thus facilitates the stratification of prognosis as well as therapy. This review summarizes the current understanding of carcinogenesis and gives a detailed overview of known morphologic and potentially future genetic prognostic parameters in malignant melanoma.

## 1. Introduction

Despite all preventive and therapeutical efforts, melanoma is still the most aggressive and deadliest skin cancer especially in persons of fair complexion. To a certain extent, primary prevention campaigns already achieved an earlier diagnosis of thinner tumors with a better prognosis [1]. Incidence rates are nonetheless increasing worldwide mainly due to unreasonable sun exposure habits, especially in young adults [2]. Once diagnosed, prognosis and therapy is stratified so far by several clinicopathological risk factors such as tumor thickness, sentinel lymph node status, ulceration, and the recently added mitotic rate [3]. In view of an often unpredictable rather heterogeneous biological behavior mainly in >4 mm thick (Stage IIC) or locally advanced melanoma (Stage III), the AJCC classification remains of limited clinical relevance in particular for these high risk patients [4]. Moreover, we currently do not have reliable tissue biomarkers that mark the disease of the individual patient for progression or complete remission [5]. At the same time, an enormous amount of basic research within the last decade has dramatically changed the molecular understanding of melanoma. Proof of several specific genomic key mutations such that BRAF could not only be causally linked to disease progression [6] but also gave rise to new, highly effective therapies targeted specifically at those mutated molecules [7]. While the multistep carcinogenesis of melanoma is still too little understood in its complexity in order to foresee when, how, and what kind of mutation develops in an invasive or metastatic tumor, genome-wide genetic analysis of primary or metastatic tumors will undoubtedly change future classifications and subsequent treatment algorithms. 

But are standard clinical prognostic parameters such as age, location, and metastasis already outdated? Could dermatopathology, the current cost-efficient diagnostic gold standard, consequently be redundant? Will we possibly be able to correlate certain histomorphologic features to specific genetic aberrations and their consecutive pathological or compensatory molecular cascades in order to recognize, treat, or even prevent the systemic metastasic impact of this tumor in our patients? These important questions arise and may contribute to a better classification of melanoma patients. With the focus on their metastatic potential, our review summarizes the current knowledge of genetic, as well as molecular features of malignant melanoma and examines their possible correlation. Moreover, we discuss the clinical implications as well as current therapies that may target these new hallmarks of melanoma.

## 2. Epidemiology of Malignant Melanoma

A growing body of evidence has already addressed melanoma as an “umbrella term” for several biological distinct subtypes as a result of multiple causative genetic aberrations, impaired pathways, or epigenetic changes. Epidemiology, in contrast, strongly indicates that UV-induced DNA damage is the primary cause of melanoma development [8], even though certain regions in which melanoma subtypes occur, such as mucosal or acral tumours, are not typically exposed to ultraviolet light. Numerous studies about phenotypic risks such as age, gender, and skin type favour sun exposure as the major cause for thinner tumors of less incidence in young patients (<35 years) on minimally exposed sites and thicker tumours in elderly patients and UV-exposed locations such as the head and neck [9, 10]. Searching for the underlying causes of initiation and progression in these melanomas, it was demonstrated that cyclobutane pyrimidine dimers (CPD) and pyrimidine-pyrimidone (PP) photoproducts are the most abundant DNA lesions in those UV-exposed tumors [11]. A well-determined repair system of minimal necessary factors such as XPA, RPA, XPC, and so forth, is, however, sufficient to remove those photoproducts from DNA [12]. Although there is clear evidence linking a deficient repair system in Xeroderma pigmentosum to a higher susceptibility of cutaneous melanoma, a presumably impaired altered expression of repair genes may also contribute to the development of melanoma but was thus far not detected [13, 14]. On the contrary, as recently shown by Gaddameedhi et al., melanoma cell lines and melanocytes have displayed an equally efficient DNA repair system in primary tumours as well as in metastasis [15]. Even in NRAS or BRAF mutant melanomas, no reduced function or expression of the DNA repair system could be found [15]. p53 mutations are only found in 1% of primary melanomas and only 5% of metastasis. Nonetheless, it was suggested that the p53-mediated repair system and well as other aberrations such as MCR1, MITF, or CDKN2A influence UV-induced expression of this potent tumour suppressor. However, it is still not known how the different p53 functions ultimately manipulate the cell fate in melanoma [15, 16]. Recent numerous molecular genetic studies, strongly support that melanomas of the trunk of younger patients with multiple nevi differ enormously from those in elderly patients with cumulatively sun-damaged skin [17, 18]. Despite the evidence for causal factors such as age, phenotype, pattern, and dose of sun exposure, the underlying genetic propensities in subentities such as desmoplastic melanoma, uvea melanoma, or melanoma in childhood are not really understood. Genome-wide studies will, however, help to identify these constitutional factors as likely heritable contributors to melanoma risk and to propose possible new target-oriented therapies in the future [19].

## 3. Clinicopathological Parameters in Malignant Melanoma

Measurable diagnostic prognostic indicators and prognostic biomarkers are needed to refine the risk and assess the outcome in patients with malignant melanoma. As much effort as has been made by the AJCC in identifying reliable risk factors, the current classification still allows only a limited stratification of this rather heterogeneous tumour [4]. Apart from the classic clinical adverse parameters such as gender, age, location, and metastasis, histopathological parameters included so far are Breslow thickness, Clark Level, ulceration, sentinel status, and the recently added mitotic rate [3]. Yet, the new forthcoming genetic features of primary tumours, for example, the BRAF or KIT mutation, are not taken into account up to now within the classifications but certainly merit reflection in the future. Although their consideration would certainly be premature, several approaches already propose to integrate those molecular markers and thereby refine distinct subcategories of malignant melanoma [18, 20, 21]. In order to identify homogeneous disease groups in greater detail and implement an improved patient management, phenotypic consequences of those genetic alterations must be better understood [22]. But in virtually all well-established, time-tested, clinicohistopathological standard factors, the underlying biological mechanisms are, as shown below, completely unknown. 

### 3.1. Breslow's Thickness

First introduced by Breslow in 1970 and later named after him, “Breslow thickness” is the eldest and one of the most important tissue biomarkers of the AJCC classification [23]. In association with horizontal enlargement, it was originally viewed and, thereafter, rectified as a parameter of tumour burden. Breslow's thickness nonetheless accurately predicts the risk of lymph node metastasis, with deeper tumours being more likely to involve the nodes [24]. Compared to Breslow's depth, Clark's level which describes the depth of tumoral penetration according to the anatomical skin layer (epidermis, dermis, and subcutis) has been proven to be less reproducible, more operator dependent, and of lower predictive value [25]. Its prognostic significance has, therefore, been limited to patients with very thin tumors in the current AJCC staging system [3]. The biological relevance of Breslow's depth's is, however, still almost unknown. Several potential molecular contributors to proliferation and, therefore, tumor thickness are currently under investigation. In particular, basic fibroblast growth factor (bFGF) is characterized as a highly mitogenic factor in melanoma especially when combined with UV [26] FGF receptor 4 (FGFR4) and its Arg388 genotype [27], cell cycle regulator proteins, or genes such as p53 and others [28, 29] as well as Bcl-2 oncoprotein [28], cell adhesion defects, or cell-cell signaling mutations [29] have proven to be correlated with increased tumor thickness. Especially for the FGFR4Arg388 allele, there was convincing evidence of intensified cell motility and invasiveness [30, 31] but also increased vertical growth and risk of metastasis in nodular and superficial spreading melanoma [27]. Even though no correlation between decreased survival rate and outcome could so far be provided and the precise mechanism is not understood, FGFR4 Arg388 polymorphism predicts a more aggressive phenotype in terms of progression in melanoma as well as breast cancer [27, 31]. As the largest genomic structure in the FGFR family, loss-of-function mutations in FGFR2 have lately also been shown to occur in subsets of melanomas [32]. Neither mutations in FGFR4 nor in FGFR2 as a possible contribution to an inherited predisposition to skin cancer, could, however, be detected in healthy caucasian women [33]. Genetic variants of FGFR4 and FGFR2 seem, therefore, to function as potential biomarkers for progression rather than as a risk factor of skin cancer development [33].

### 3.2. Ulceration

In contrast to an ulcer due to trauma, ulceration in melanoma is defined as “a consumption of the epidermis” with a thinned epidermis to the side of the defect [34]. Initially identified as an adverse prognostic parameter by Allen and Spitz in 1953 [35], subsequently validated by Balch et al. [36], and later on by numerous other studies [37–39], ulceration has been convincingly shown to be an independent predictor of sentinel status and overall survival even in high-risk thick melanomas >4 mm [40, 41]. Despite its inclusion in the AJCC classification already in 2001 [42], the knowledge about why, when, and for what reason ulceration occurs and how it favours tumor progression is at best theoretical. Studies concentrated on width [36], depth [43], and proportion of ulceration [44], its association with mitotic rate [45] or vascular involvement, and tumor vascularity [46]. The results were, however, often inconclusive. The most plausible hypothesis that considered ulceration as a consequence of tumor proliferation, and therefore secondary epidermal thinning and contact ulceration, has been reevaluated. A recent study has demonstrated an independent prognostic association of ulceration and mitotic activity [47]. In addition, a direct influence on the local tumor environment seems nonetheless possible. Hence, ulceration challenges the control functions of keratinocytes, melanoma cells are enabled to transform more easily, therefore favoring tumor progression [48].

### 3.3. Regression

More common in melanoma than in any other neoplasia [49], regression is defined as a partial or complete disappearance of the tumor without treatment [50]. Due to the loss in pigmentation in terms of a blue or grey-whitish discoloration, it is clinically highly apparent in this particular tumor entity. With an incidence of approximately 10–35% of patients with primary malignant melanoma [51], regression arises specifically in thinner tumors but hardly ever in nodular melanoma [52]. Associated with variable degrees of inflammatory and stromal changes, this particular phenomenon proceeds from an early dense lichenoid infiltrate of lymphocytes and dermal edema to a late fibrosis and a usual melanosis within a thickened papillary dermis [53]. Especially when the tumor is pigmented, melanophages as the histopathological telltale sign are often present. Although the current understanding of regression is clearly that of an immune-mediated, cancer-autonomous process [21], neither its biological significance nor the underlying molecular or genomic aberrations are so far recognized. Possible explanations vary from an increased T-cell response [54], an inhibited angiogenesis [53], to a forced apoptosis of tumor cells [53, 55]. Consequently there are different therapeutical implications of regression. While a positive host immune response may supersede wider excision margins or sentinel lymph node biopsy [56, 57], regression may, however, on the other side indicate a formerly deeper infiltrating tumor and thus a lower threshold for sentinel lymph node biopsy [58]. Especially in thin melanomas <1 mm, regression as a left-over of a presumably thicker tumor therefore still leads to wider surgical margins and a lower threshold for SLN biopsy [58]. The most convincing, although unproven, hypotheses for a regression-driven tumor progression so far are the Hammon's effect, which postulates a natural selection of aggressive residual tumor clones as a result of regression [59, 60] and Bastian's telomere crisis, which argues that a massive senescence and cellular apoptosis equally favor the selection of genomic aberrations and therefore progression [55]. Future epidemiologic studies investigating the impact of regression of the primary tumor for the prognosis of melanoma are certainly required to further investigate those intriguing details.

### 3.4. Mitotic Rate

Tumor proliferation as defined by mitotic rate has been confirmed as an independent adverse prognostic parameter in many solid neoplasia including melanoma [61–64]. Due to the fact that its increase is significantly correlated with reduced survival rates primarily within melanoma of less than 1 mm tumor thickness, it has recently replaced Clark's level as the primary criteria for defining the subcategory of T1b in AJCC classification 2009 [3, 65]. The lack of a universally agreed approach of how to document mitotic figures led to many studies that did not include mitotic rate in their analyses up to now [66]. As recently detailed by the AJCC manual, starting with dermal areas that contain most mitoses (so-called hot spots), and extending the approach later to adjacent fields up to 1 mm^2^, now allows for the first time a reproducible assessment [3] although this approach is time consuming to the dermatopathologist. So far, only two sorts of genes and their pathways are identified to be overrepresented in melanoma with higher mitotic activity. Replication Origins Firing (ROF) genes such as MCM4 and MCM6 as well as the oncogene securin are strongly correlated with metastases and therefore poorer prognosis even after considering other prognostic parameters such as sex, age, location of the primary, thickness, and ulceration [29, 67]. As much effort has been made in defining the biological relevance of these dermatohistopathological parameters, they cannot reliably distinguish the metastatic behaviour of certain subgroups such as Stage IIC melanoma. Moreover, the exact diagnosis in some cases of melanoma might be problematic altogether as the individual assessment of these criteria differs among pathologists [68]. In addition, benign melanocytic proliferations such as atypical nevi can also display a number of those features, given that routinely performed immunohistochemical markers, for example, S100B and HMB-45 are of little help in distinguishing nevi from melanoma [69]. Taken these reflections into account, a more molecular understanding of melanoma might therefore be desirable. Inevitably, the understanding of the molecular basis of malignant melanoma has to be further improved to identify the critical “drivers” and “passengers” during oncogenesis of melanoma [70, 71].

## 4. Current Knowledge about Oncogenesis of Malignant Melanoma

**Figure 1:**
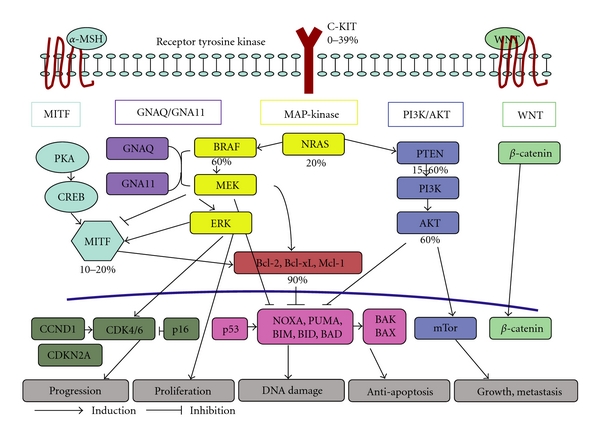
Signaling pathways in malignant melanoma (modified according to http://www.cancercommons.org/).

The core issue obscuring the best possible treatment of malignant melanoma is still its unpredictable pattern of progression and metastasis. Well-established prognostic parameters alone or in combination are so far not effective enough to accurately predict the outcome for every individual patient. Biologically distinct as malignant melanoma is, the greatest therapeutical potential lies without doubt in the understanding of what key indicators influence the course of the disease most, regardless whether they may be genetic, possibly molecular, least likely clinical, or even combined, and therefore predispose for the risk of systemic disease. The multistep process of carcinogenesis in malignant melanoma is, however, complex and at best only in part understood. A number of excellent reviews have summarized the exciting developments in the understanding of this tumor in depth [72]. To date, four pivotal, nonlinear, and rather netlike interwoven defective signaling pathways have been implicated. These are MAP kinase, PI3K/AKT, MITF, and WNT. The following scheme gives a simplified overview of these pathways with their most common aberration and the percentage of mutations detected within these signaling pathways. Certain rare subtypes such as uveal melanoma also have been found to have mutations in GNAQ [73] or GNA11 [74] that also lead to constitutive activation of these signaling pathways (Figure 1). 

Proven to be one of the most frequently mutated cascades in melanoma, the mitogen-activated protein (MAP) kinase pathway shows several pathologically activated mutations that may contribute to malignant transformation. The most common mutations or cytogenetic amplifications occur in the BRAF, the KIT, the NRAS, or the CDKN2A genes. In 8–12% of familiar malignant melanoma alone, mutations of CDKN2A gene that are linked to chromosome 9p21 arise [75, 76]. 

Unlike regular sites of cutaneous melanoma, uncommon subsets of melanocytic neoplasia such as uveal melanoma or malignant blue nevus lack frequent oncogenetic mutations in cKIT, NRAS, or BRAF [77–79]. Notwithstanding other oncogenes such as the alpha subunit of a class of heterotrimeric GTP-binding proteins (Gq), namely GNAQ and GNA11, are activated. Hypermorphic mutations in those genes were found to contribute to skin darkening and therefore melanocyte biology in mice [80]. Proven to occur early in progression, they seem, however, not to be related to clinical outcome so far [81, 82]. When active, GNAQ and GNA11 alternatively upregulate the MAP kinase pathway [73]. Operating downstream of several G-protein coupled receptors, GNA11 has presumably a more potent adverse effect than GNAQ in locally advanced or metastasized tumors although overall survival did not differ [74]. GNAQ mutations are, however, considered to be more sensitive to the upcoming therapeutical MEK inhibition [73].

Cross-linked via NRAS, the MAP kinase cascade also initiates the PI3K and thereby the PI3K signaling pathway, another defective cascade found in a large percentage of melanomas. Apart from NRAS, either deletion of PTEN or overexpression of AKT mainly lead to the stimulation of mTOR, a central regulator of cell growth and proliferation that has raised substantial interest in this signaling pathway in melanoma [83]. 

Of central importance for benign as well as malignant melanocytes, MITF and its cascade were found to represent a central transcription factor that regulates differentiation in the pigment cell system [84]. In addition to *α*-MSH and ACTH that activate MITF via the MC1R, it is also physiologically regulated by MAP kinase and PI3K signaling pathway [85, 86]. In the development of melanoma, however, an optimized level of MITF as an oncogene for proliferation and survival of tumor cells needs to be maintained by BRAF [87]. Insufficiently high or low expression of MITF results in tumor-protective differentiation, cell cycle arrest, and subsequent apoptosis [88]. MITF amplification, single based MITF substitution and even mutation of its regulator SOX10 have all been proven lately to be causative for altered MITF function in both primary and metastatic melanoma [89, 90] underscoring the involvement of MITF in melanomagenesis.

Although mutations of the *β*-catenin gene and APC have already been detected, the WNT signaling pathway has not been extensively implicated in melanoma development this far, due to the fact that defective *β*-catenin is rarely identified although it clearly acts as a melanoma-specific antigen [91, 92]. Under physiological conditions, WNT-signaling proteins bind to Frizzeled receptors, thereby stabilizing *β*-catenin with subsequent release from a multiprotein complex. It then accumulates in the nucleus and initiates as a coactivator the transcription of a multitude of target genes. In case of genetic mutations of *β*-catenin, such as in malignant melanoma, it forms a complex with LEF-1 (lymphoid enhancer-binding protein), which in turn leads to malignant transformation of the cell [93, 94]. In particular Wnt-2, a survival factor in human carcinogenesis, [95] has lately become focus of intensified research as a biomarker and a potential target to subclassify and treat malignant melanoma [96, 97]. Besides the main canonical WNT signaling pathway, a variation of the so-called noncanonical pathway with altered receptors and enzymes, and even a signal regulated in a paracrine manner (the so-called “notch” cascade), diversify and complicate the WNT signaling pathway considerably [98]. Specific inhibitors in terms of small molecular antagonists or RNA aptamers have nonetheless been developed to potentially target this pathway, an intruiging possibility given the important role of this pathway in the so-called “tumor-initiating cells” in other tumor entities [99]. WNT2 has also been found to be overexpressed in malignant melanoma [97]. Of therapeutic interest, a specific anti-WNT-2 monoclonal antibody has been proven to inhibit WNT signaling and subsequently induce apoptosis [96]. 

The complexity of these crosstalking circuits is increased even more by the fact that one genetic alteration is not enough to make a melanoma. Several additional changes are needed in a multistep process to result in malignant transformation [100]. Cumulative genetic instability gradually induces arbitrary genomic aberrations that lead to uncontrolled replication and growth, inhibition of apoptosis, and finally the ability to invade and metastasize due to a Darwinian-like selection process of the tumor cells [101–103]. Considering further stem cell-determined, epigenetic, tumor-environmental, or immunologic changes, the variety of possible influencing factors on the classic hallmarks of cancer is multiplied beyond measure [104], and the knowledge of critical constitutional and somatic genetic parameters is not yet complete [22].

## 5. Epigenetics

Recent progress in the understanding of genetic aberrations in malignant melanoma has likewise prompted significant efforts in defining so called “epigenetic” changes that accompany the malignant transformation in melanocytes. Defined as any changes in gene expression that are not achieved through alterations in the primary sequence of the genomic DNA, epigenetics influence a wide range of alternative gene functions such as cell cycle regulation, cell signaling, differentiation, DNA repair, apoptosis, invasion, metastasis, angiogenesis, and immune recognition [105]. Although their precise contribution to tumor progression is still unknown, they were proven to efficiently restore the expression of aberrantly silenced genes and thereby to reestablish silenced signaling pathway function [106]. The most clearly identified epigenetic mediators so far are the methylation of DNA in the context of CpG dinucleotides, the posttranslational changes of histone proteins and, though less characterized, the influence of microRNAs (miRNAs). The reactivation of “sleeper” genes and the maintenance of these epigenetics aberrations requires functioning enzymes such as DNA methyltransferases (DNMT) or histone deacetylases (HDAC), and histone methyl transferases (HMT), respectively. In case of DNA methylation, three different DNMTs are implicated in new methylation patterns with gene-specific hypermethylation on the one hand as well as genome-wide hypomethylation on the other [107]. In addition to genetic alterations, epigenetic DNA hypermethylation is, therefore, a complementary, frequent, and important mechanism to inactivate tumor suppressor genes such as CDKN2A [108]. While hypermethylation silences tumor suppressor genes, global hypomethylation might, however, activate the expression of oncogenes. This could lead to a diversified and significantly impaired methylation disbalance of multiple genes that eventually initiates genomic instability, tumorigenesis, and cancer progression [106, 107]. As common as this phenomenon of hypomethylation is in many tumors, little is known so far about target genes regulated by this event in melanoma [109]. Similar to the discussion of driver and passenger mutations in genetic aberrations, the biological significance of several identified aberrantly hypomethylated epigenetic genes, for example, cancer-testis antigen (CTAs), PRAME, and MAGE continue to be poorly understood. Nonetheless, given the broad relevance of these pathways in almost every tumor entity, substances have already been developed for therapeutical approaches, and the epigenetic status of certain genes may potentially predict the biological function and could serve as a biomarker [110, 111].

Along with DNA methylation patterns, initial studies about histone acetylation have addressed a possible role in melanoma development and progression [112]. In particular hypoacetylation-mediated downregulation of CDKN1A and, similarly, proapoptotic proteins such as BAX, BAK, BID, and BIM may profoundly influence cell cycle and apoptosis of the cell and thereby lead to tumor progression or therapeutic resistance [113, 114]. In the demanding packing and outpacking machinery of genomic DNA into nucleosomes and chromatine, respectively, at least three groups of histone acetyltransferases (HAT) and 18 identified histone deacetylases (HDAC) are involved thus far [115]. Complicating this picture, histone methyl transferases (HMTs) modulate the chromatin compaction grade of the DNA that finally determines the transcriptional status of target genes [116]. In contrast to DNA methylation, the knowledge of the posttranslational aberration of histones is altogether scarce and mainly gathered indirectly by treatment results of HDACs thus far. Promising results of multiple HDAC inhibitors concerning vascular endothelial growth factor (VEGF), generation of reactive oxygen species (ROS), cell death, senescence, and especially intrinsic as well as extrinsic apoptosis in the transformed cells have already been described in various solid tumor entities [117–119]. Proapoptotic stimuli are, however, known to be less effective in human melanoma cell lines. Recently discovered key mediators such as the cleavage of Poly-ADP ribose protein (PARP) [113] and HDAC inhibitors like the short fatty acid VPA [120] have led already to promising results with antitumor activity in combination therapy with anthracyclines in melanoma [121]. The level of understanding of the molecular mechanism in histone posttranslational modifications has yet to become more refined to predict the outcome of such promising therapies in subgroups or individual melanoma patients.

The most recently discovered players in epigenetic regulation have been noncoding microRNA (miRNA). Once transcribed in the nucleus and further processed by several intermediate stages, they are finally incorporated into a RNA-induced silencing complex that recognizes their target miRNA. This either inhibits their translation or (less frequent) causes their degradation [122]. Each miRNA has several target RNAs and vice versa. In addition to more than a hundred currently confirmed miRNAs, more than 1000 miRNA have been predicted by bioinformatics [123]. Despite the limited data available so far, miRNAs are proven to play pivotal roles in the epigenetic pathogenesis of human cancer. As proof of principle, several key miRNAs have already been identified in driving tumorigenesis and progression in malignant melanoma [124]. Especially the lack of an inhibition by miR-137 and miR-182 was found to result in an overexpression of MITF, a master regulator in benign melanocytes as well as melanoma [124]. On the other hand, overexpression of miR-182 contributes likewise to progression and metastasis by repressing MITF [124]. In a similar way, miR-34b, miR34c, miR199a, and miRNAs involved in the expression of the oncogene MET modify target gene expression in accordance with the stage of cancer development [125]. Considering the fact that miRNAs themselves are also targets of epigenetic regulations as, for example, miR-34a, which is proven to be silenced by a CpG-mediated methylation in up to 60% of primary melanomas [126], further studies are mandatory to define their role in melanoma biology more precisely.

## 6. Oncogene-Defined Targeted Therapy in the Era of BRAF Inhibitors

As one of the most devastating forms of cancer in terms of life expectancy and outcome, metastatic melanoma was until recently an almost intractable disease. This was largely explained by the fact that mono- or polychemotherapy, the standard of care for over 30 years, only benefits a very small subset of patients. With the discovery of an activating mutation of BRAF in 50–60% of all melanoma, with 90% of these tumors carrying a substitution at V600, a first tumor-specific target for a treatment was identified in 2002 [6]. Sorafenib, a multikinase inhibitor and one of the first targeted therapies in clinical testing, has unfortunately shown little efficacy in patients with activated MAP kinase pathway (and therefore BRAF positive) patients [127]. Consequently, more selective BRAF inhibitors were subsequently tested in clinical trials, which in case of vemurafenib (also known as PLX 4032) and GSK2118436 have demonstrated unprecedented clinical results in metastatic malignant melanoma harboring BRAF mutation [7, 128, 129]. Within two weeks, the majority of patients stated a symptomatic improvement, and approximately 60% showed an objective response according to response evalutation criteria in solid tumors (RECISTs). Overall about 80% of all patients with metastatic tumors experienced some degree of regression [7]. In the subsequent extension phase of the trial, 81% patients demonstrated tumor regression, and the progression-free survival was at an average of 7 months [7]. Dose-dependant adverse events like rash, photosensitivity, fatigue, and arthralgia were well managed by either dose reduction or by the termination of the treatment if necessary. GSK2118436 has proven to be even of higher potency at a lower concentration [129]. Apart from pyrexia, rash, fatigue, headache, nausea, and vomiting, severe adverse events such as squamous cell carcinoma and keratoacanthoma were reported. A series of publications, however, quickly discovered novel mechanisms that paradoxically activate the MAP kinase pathway in the presence of BRAF inhibitors [130, 131]. Due to three isoenzymes of RAF (A-RAF, B-RAF, and C-RAF), the inhibition of one of them such as B-RAF can induce a compensatory transactivation of C-RAF, which in turn activates downstream MEK and the subsequent pathway [130, 132]. As a consequence of “gatekeeper” mutations that sterically prevent the inhibitor binding to the active side in RAF, the crossactivation of C-RAF is not always initiated and even to a certain extent inhibited by the given drug [132]. ATP competitive inhibitors for instance are supposed to stabilize the interaction between B-RAF and C-RAF [133]. Besides C-RAF as a paradoxical bypass of B-RAF, other ERK-dependent mechanisms such as N-RAS mutation, COX overexpression, or MEK1 mutations contribute to an acquired resistance to B-RAF [134]. Complicating the picture, even ERK-independent alterations like PDGFR*β* overexpression, IGF1R activation and PTEN loss have been identified to reactivate ERK signaling in B-RAF mutant tumors [134, 135]. Although the benefit of B-RAF inhibition as monotherapy has been sufficiently confirmed, rapidly occuring secondary resistance mechanisms in tumors will most likely favor combination therapies targeting other genetic “hot spots” in melanoma such as MEK, RAS, and KIT.

RAS, in particular N-RAS mutations, occur in approximately 15–25% of malignant melanoma. They inhibit the GTPase-mediated activity of RAS and thus keep it in an continuously active state [136]. Demanding as task to develop an agent is that would rival GTP, several interacting pathways such as MAP kinase or PI3 kinase seem to play an important role in the N-RAS mutant subset of melanoma [137, 138]. Mutually exclusive to B-RAF V600E mutation [136], NRAS mutations have been shown to be sensitive to MEK-targeted therapies particularly in combination with PI3K, AKT, or mTOR inhibitors [137]. 

KIT mutations have so far been found in a small subgroup of melanomas, in particular acral or mucosal tumors that are not related to sun exposure [20]. According to the results in gastrointestinal stroma tumors (GISTs), KIT inhibitors such as imatinib and sunitinib, and newer inhibitors such as nilotinib or dasatinib have been described, however, to be less responsive [139, 140]. Encouraging to this subgroup of patients, anecdotal reports have shown complete remission lasting up to one year [141]. 

Despite several promising new agents (Table 1), there are, however, still no therapeutic strategies that would reliably conquer the complexity of pathways resulting in a highly aggressive malignancy in melanoma. Considering several multimarker assays using in vivo samples and cell culture of primary melanomas and metastasis together, melanoma development itemizes to several hundreds of involved genes that seem too plentiful to be individualized for a targeted therapy in a single patient, even though new, potentially essential, marker genes have been identified and are currently tested [142]. The very view of resistance, unwanted side effects, and rapid progression after initial responsiveness clearly emphasize the importance of a thorough, genotypical stratification, and a “driver-focused” synergistic therapy. The development of an oncogene hierarchy with differentiation into important drivers and bystanding passengers seems therefore necessary.

## 7. Conclusion

The recently gained knowledge about the functional importance of muted genes in a high proportion of malignant melanoma has fundamentally changed the diagnostic and therapeutic approach. In view of the focus on BRAF, NRAS, KIT, and PTEN, four key genomic defective alterations and their corresponding pathways are identified that without any doubt refine and extend the understanding of its bewildering biological complexity. Although an improved classification [4, 18, 22] and corresponding risk stratifications and target-oriented therapies (Table 1) are within reach, or in case of the latter even under effective investigation, a restriction to some precious few control factors seems to be a too easy answer. The serious question remains, how do the highly relevant histopathological parameters translate in a benefit for distinct subsets of the melanoma patients? 

The answer probably lies in the identification of the biological “Achilles heel” of individual tumors. As convincingly shown, molecular analysis of subsets of melanoma has at first revealed mutations in cKIT. This knowledge was then rapidly translated into a successful targeted therapy [18]. Other positive examples are the more recent successful translation of the knowledge of the BRAF mutational status (e.g., V600E) into elegant mutation specific, and at least short-term successful therapy in these patients [7]. However it is not surprising that in a large number of melanoma patients such single mutations do not precisely delineate the biological behaviour of the tumor at the time of primary melanoma diagnosis. In fact, there appear to be a multitude of biologically distinct melanoma entities. Thus, it is likely that this straightforward approach is too narrow, given that in a considerable fraction of melanomas so far unknown oncogenes or tumor suppressors, or combinations thereof may control tumor cell fate [143]. Most likely unbiased approaches to melanoma using 21st century technology of genetic profiling will yield intriguing results [144]. As much as the classic hallmarks of cancer withstood the test of time [102]: recently discovered characteristics such as antiapoptotic parameters [145], the role of tumor stem cells [146], telomerases [147], or circulating tumor cells [148], as well as other tumor-environmental and epigenetic phenomena [106, 115] have also to be taken into account and may translate into successful therapy [104]. But hopefully, as Hanahan and Weinberg lately stated, this phenotypic myriad in melanoma [19, 149] may portray just a few of the causal principles of distinct tumor cell types that need to be clarified in order to improve the treatment and outcome in our melanoma patients [104]. So, in the era of molecular profiling, the gist of the matter “what's really risky in melanoma” seems within reach.

## Figures and Tables

**Table 1:** Genetic mutations and corresponding current and future targeted therapies.

Pathway	Target	Therapy
MAP-kinase	Receptor tyrosine kinase	Imatinib
		Dasatinib
		Nilotinib
		Masitinib
	BRAF	GSK2118436
		Vemurafenib
	NRAS	Sorafenib
		Tipifamib
	CRAS	Lonafamib
		RAF265

PI3AK	mTor	Sirolimus
		Temsirolimus
		Everolimus
	PI3, AKT	GDC0941, GSK2126458, BEZ235, BKM120, XL765, MK2206, GSK 690693

MITF	CDK2, HDAC	SCH727965, panobinostat

WNT	B catenin	Small molecular antagonists
		RNA aptamers

## List of Abbreviations to Figure 1

*α*-MSH: Ligand of melanocyte-stimulating hormone
*β*-Catenin: Tumor-oncogene ind Wnt pathway
BAK/BAX: Proapoptotic effectors of Bcl-2 gene family
Bcl-2/Bcl-xL/Mcl-1: Proapoptotic members of Bcl-2 gene family
BIM, BID, BAD: Proapoptotic members of Bcl-2 gene family
BRAF: Serine/threonine-protein kinase B-Raf protooncogene
CCND1/CDK4/6/CDKN2A: Cyclin-dependent kinases (CDKs)
c-Kit: Protooncogene
ERK: Extracellular-signal regulated kinase
GNAQ: Guanine nucleotide-binding protein G(q) subunit alpha
GNA11: Guanine nucleotide-binding protein subunit alpha-11
MAP/MEK: Mitogen-activated proteins
MITF: Microphthalmia-associated transcription factor
mTOR: Mammalian target of rapamycin
NOXA/PUMA: p53-inducible proapoptotic members of the Bcl-2 family
NRAS: Neuroblastoma RAS viral oncogene homolog
P16: Cell cycle regulator
P53: Tumor suppressor gene
PI3K: Phosphoinositid-3-kinase
PTEN: Phosphatase tensin homolog, tumor suppressor
WNT: Ligand of WNT-pathway.
